# Supplementary material for: Oral Squamous Cell Carcinoma Contributes to Differentiation of Monocyte-Derived Tumor-Associated Macrophages via PAI-1 and IL-8 Production
Source: Int J Mol Sci. 2021 Aug 31;22(17):9475. doi: 10.3390/ijms22179475 (PMC8430735; doi:10.3390/ijms22179475)
Supplement: Supplementary file 1 [file ijms-22-09475-s001.zip › ijms-1331942-supplementary.pdf]

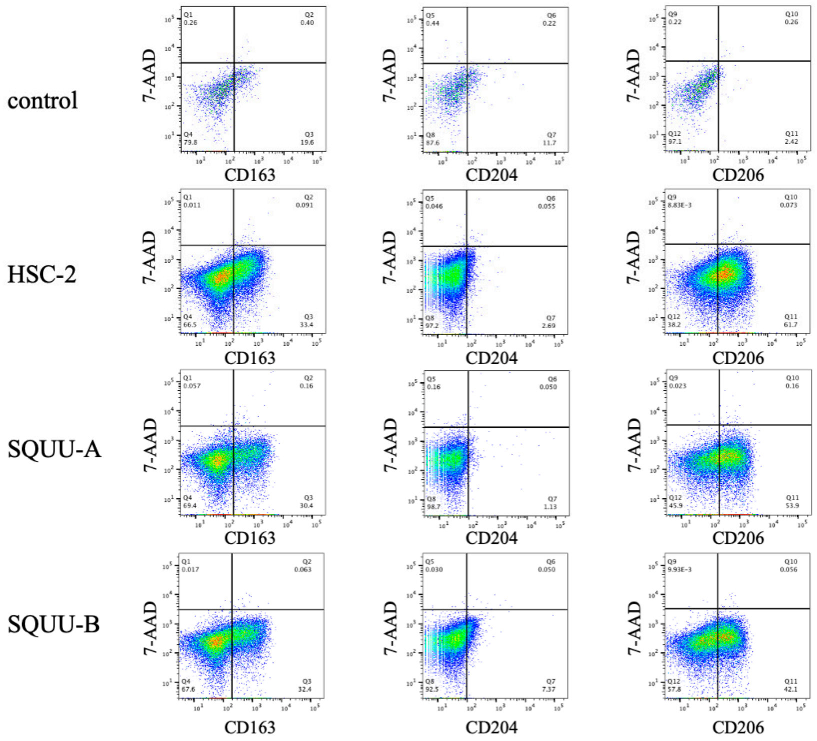

Supplementary Figure S1: Representative image for the co-culture of 7-AAD⁺TAM subsets co-cultured with OSCC cell lines (HSC-2, SQUU-A, and SQUU-B cells) for 4 days. The detailed methods for cultivating cells are described in the Materials and Methods section

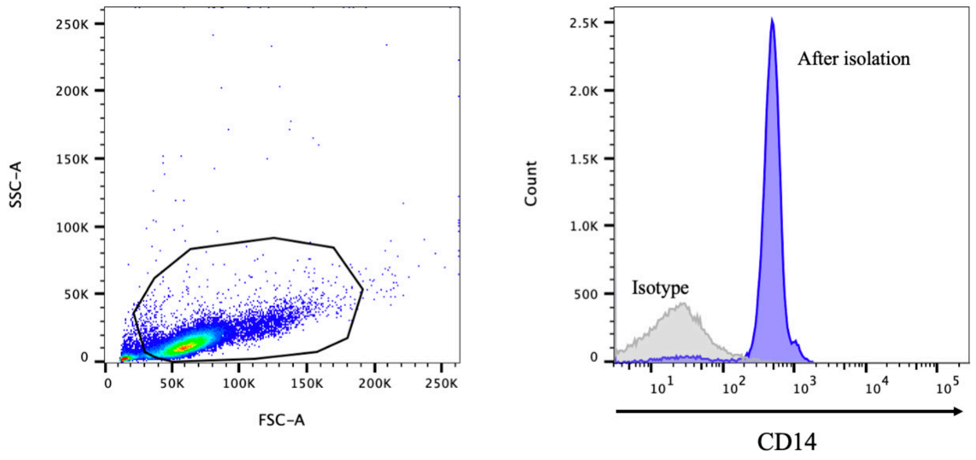

Supplementary Figure S2: Purity of the isolated CD 14⁺ cells were as determined by flow cytomemetric analysis. The detailed methods for isolating cells are described in the Materials and Methods section.

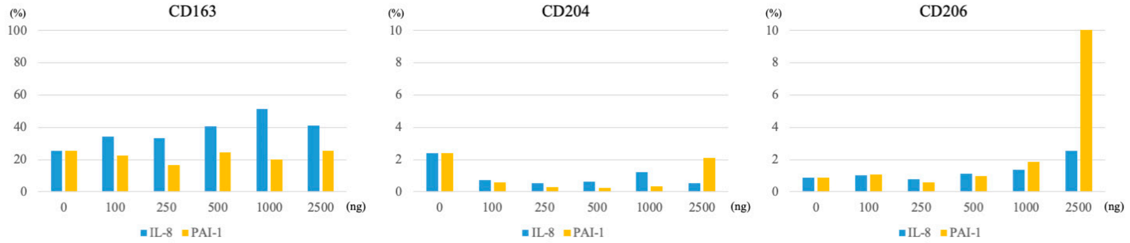

Supplementary Figure S3: Positive rate of CD163, CD204, and CD206 in $CD14^+$ monocytes stimulated with IL-8 or PAI-1, as determined by flow cytomemetric analysis.
